# How Are Epigenetic Modifications Related to Cardiovascular Disease in Older Adults?

**DOI:** 10.3390/ijms22189949

**Published:** 2021-09-14

**Authors:** Mojgan Gharipour, Arya Mani, Mona Amini Baghbahadorani, Camila Kellen de Souza Cardoso, Shayesteh Jahanfar, Nizal Sarrafzadegan, Cesar de Oliveira, Erika Aparecida Silveira

**Affiliations:** 1 Isfahan Cardiovascular Research Center, Cardiovascular Research Institute, Isfahan University of Medical Sciences, Isfahan 8158388994, Iran; gharipour@crc.mui.ac.ir; 2 Cardiovascular Research Center, Department of Internal Medicine, and Department of Genetics, Yale University School of Medicine, New Haven, CT 06520, USA; arya.mani@yale.edu; 3 Interventional Cardiology Research Center, Cardiovascular Research Institute, Isfahan University of Medical Sciences, Isfahan 8158388994, Iran; amini.mona1995.ma@gmail.com; 4 School of Social Sciences and Health, Nutrition Course, Pontifical Catholic University of Goias, Goiânia 74605-010, Brazil; camilacardoso_nut@hotmail.com; 5 Department of Public Health and Community Medicine, Tufts University School of Medicine, Boston, MI 02111, USA; shayesteh.jahanfar@tufts.edu; 6 Faculty of Medicine, School of Population and Public Health, The University of British Columbia, Vancouver, BC V6T 1Z3, Canada; 7 Department of Epidemiology & Public Health, Institute of Epidemiology & Health Care, University College London, London WC1E 6BT, UK; c.oliveira@ucl.ac.uk; 8 Graduate Program in Health Sciences, Faculty of Medicine, Federal University of Goiás, Goiânia 74690-900, Brazil

**Keywords:** epigenetics, older adults, cardiovascular disease, aging, lifestyle, environment, physical inactivity, diet, nutrients, caffeine, alcohol consumption

## Abstract

The rate of aging has increased globally during recent decades and has led to a rising burden of age-related diseases such as cardiovascular disease (CVD). At the molecular level, epigenetic modifications have been shown recently to alter gene expression during the life course and impair cellular function. In this regard, several CVD risk factors, such as lifestyle and environmental factors, have emerged as key factors in epigenetic modifications within the cardiovascular system. In this study, we attempted to summarized recent evidence related to epigenetic modification, inflammation response, and CVD in older adults as well as the effect of lifestyle modification as a preventive strategy in this age group. Recent evidence showed that lifestyle and environmental factors may affect epigenetic mechanisms, such as DNA methylation, histone acetylation, and miRNA expression. Several substances or nutrients such as selenium, magnesium, curcumin, and caffeine (present in coffee and some teas) could regulate epigenetics. Similarly, physical inactivity, alcohol consumption, air pollutants, psychological stress, and shift working are well-known modifiers of epigenetic patterns. Understanding the exact ways that lifestyle and environmental factors could affect the expression of genes could help to influence the time of incidence and severity of aging-associated diseases. This review highlighted that a healthy lifestyle throughout the life course, such as a healthy diet rich in fibers, vitamins, and essential elements, and specific fatty acids, adequate physical activity and sleep, smoking cessation, and stress control, could be useful tools in preventing epigenetic changes that lead to impaired cardiovascular function.

## 1. Introduction

The World Health Organization (WHO) predicts that the worldwide proportion of individuals aged 65 and older will double between the years 2000 and 2050, from the current 6.9% to 16.4% [1]. The older adult population in 2050 will be 16 times larger than that in 1998, with the male-to-female ratio of centenarians falling to almost 1:4 [2]. Although increasing the population’s longevity is considered an accomplishment of healthcare systems, aging has been associated with a progressive decline in several physiological processes and increased risk of chronic conditions [3]. Aging increases the burden and prevalence of many common diseases such as cancers, diabetes, and cardiovascular disease (CVD). CVD is the leading cause of death later in life [4]. Aging affects the cardiovascular system as reflected in pathological conditions such as myocardial hypertrophy, changes in left ventricular (LV) diastolic function, diminished LV systolic reverse capacity, increased arterial stiffness, and impaired endothelial function. For example, increasing arterial stiffness leads to myocardial compensatory mechanisms, including LV hypertrophy and fibroblast proliferation, resulting in decreased cardiac output and an increase in fibrotic tissue [5,6,7]. Older adults frequently suffer from various comorbidities and often they have different comorbidities, therefore determining several phenotypes. These various phenotypes are characterized by different frailties as well as different cardiovascular outcomes. Findings from the Registro Politerapie SIMI (REPOSI) registry showed that being male, history of hospitalization, polypharmacy (more than five drugs), lower functional status and fragility, depression, CVD, chronic obstructive pulmonary disease, and urinary tract infection were related with a higher risk of hospitalization in older adults [8]. Moreover, aging is characterized by chronic low-grade systemic inflammation, and is associated with multiple chronic conditions such as ischemic heart disease, heart failure, myocardial infarction, diabetes, lung cancer, osteoporosis, and metabolic syndrome as well as CVD risk factors such as lipid disorders [9,10,11,12]. A study by Li et al., demonstrated that an epigenetic modification in ELOVL fatty acid elongase 2 (Elovl2), a gene whose epigenetic alterations are most highly correlated with age prediction, contributes to aging by regulating lipid metabolism. Impaired Elovl2 function disturbs lipid synthesis with increased endoplasmic reticulum stress and mitochondrial dysfunction, leading to key accelerated aging phenotypes [13]. 

Consequently, understanding the molecular mechanisms related to CVD throughout the life course could help us to understand what happens during aging in the cardiovascular system. A growing body of evidence suggests that epigenetic modifications may significantly disrupt gene expression routes during the life course, thus affecting the molecular phenotype and function of involved cells [14]. Therefore, in the present review, we discuss the emerging role of epigenetics in CVD among older adults and the potential impact of lifestyle and environmental modifications on preventing deleterious epigenetic changes in this age group [15]. 

### Aging, Cardiovascular Disease, and Epigenetics

Aging results from the collective effect of molecular and cellular damage over time [16]. A combination of genetic and environmental factors (e.g., diet, smoking, obesity, and stress) affects the aging process [16]. At the molecular level, any changes in gene expression can result in altered cellular and tissue function. For example, aging of the heart is accompanied by changes in the expression of genes encoding proteins that are involved in inflammatory and stress responses that, when exceeding the homeostatic levels, impair cardiac function [17]. These changes can be triggered by genetic mutations or epigenetic modifications that cause changes in the gene expression profile. Any changes in the structure of DNA, RNA, and proteins throughout life alter their function and may lead to changes in cellular and organ function, leading to various diseases [18,19,20]. Consequently, understanding the molecular processes that contribute to CVD during the life course could provide information on the mechanisms that underlie cardiovascular aging. In the present review, we discuss the emerging role of epigenetics in CVD among older individuals and the possible impact of lifestyle and environment in this population [15].

## 2. Aging, CVD, and Epigenetic Modification

While cardiac hypertrophy and cardiac fibrosis are considered as the main causes of heart failure, several genes have increased expressions, e.g., Nappa, Nppb, Myh7, and skeletal alpha-actin [19,20,21].

### 2.1. Age-Related DNA Methylation

DNA methylation, an epigenetic mechanism that leads to changes in gene expression, is heritable and impacted by aging and various environmental exposures [22]. Levels of DNA methylation, which cluster in specific loci of the human genome, could be used as a marker of biological aging. DNA methylation age (DNAmAge) can provide an estimate of the biological age, and can be used as a tool to estimate “accelerated biological aging” which contributes to several diseases such as diabetes, CVD, and dementia, and, ultimately, mortality risk [20,23,24,25,26,27,28,29,30]. A recent methylome-wide association study conducted on 718 men and women aged between 25 and 72 demonstrated a positive correlation between increased methylation of CpG islands, shores, and shelves with aging [25,31]. Numerous studies have associated DNA hypermethylation with the pathogenesis of atherosclerosis [32,33]. 

Interestingly, a monozygotic twin study demonstrated that the epigenomes of young monozygotic twins are very similar, but patterns of methylation in monozygotic pairs differ as they age [25]. In this regard, Fraga et al., examined the global and locus-specific differences in DNA methylation and histone acetylation of a large cohort of monozygotic twins and demonstrated that while twins are epigenetically indistinguishable during the early years of life, they exhibited noticeable differences in their overall content and genomic distribution of 5-methylcytosine DNA and histone acetylation as they aged, resulting in overall different gene expression profiles [25]. Specific methylation changes, usually hypermethylation, have been found in the promoter region of genes that are considered protective against atherosclerosis, such as extracellular superoxide dismutase, estrogen receptor α, endothelial nitric oxide synthase, and 15-lipoxygenase [34,35]. McKay et al., identified the genome-wide DNA methylation changes and the locus-specific CpG alterations taking place during the onset and progression of human atherosclerotic lesions. The methylation of the p66Shc promoter is reduced by different CVD risk factors (i.e., hyperglycemia, ox-LDL), whereas JunD promoter methylation is increased, the latter being particularly evident with aging [36]. Both p66Shc and JunD expression levels are profoundly altered in the circulating endothelial progenitor cells isolated from older adult patients compared to young individuals [37]. 

### 2.2. The Effect of Aging on Histone Modifications

The basic unit of chromatin structure is the nucleosome, which consists of 147 base pairs of DNA wrapped around a histone octamer that comprises two copies of each core histone protein, H2A, H2B, H3, and H4 [38]. The post-translational changes in histone tails are another epigenetic modification that regulates gene expression by chromatin remodeling. 

Histone methyltransferases are responsible for the methylation of histone lysine and arginine at different sites. Briefly, H3K4 methylation induces gene activation while H3K9 and H3K27 methylation inhibit gene expression. In addition, modification of polymethyl groups on histone lysine leads to different levels of methylation that may have different biological significance. Interestingly, age-related DNA hypermethylation in mesenchymal stem cells (MSCs) is associated with repressive histone marks H3K27me3/H3K9me3 [39], while hypomethylated DNA sequences are powerfully enriched with the active chromatin mark H3K4me1 [40]. Histone modifications such as acetylation of histone 3 at lysine 9 (H3K9Ac) and trimethylation of sirtuins are crucial regulators of the aging process from yeast to mammals [41].

A study carried out by Ashleigh et al., demonstrated that changes in histone modifications facilitated the development and progression of carotid atherosclerotic plaques of patients with carotid artery stenosis. They indicated that the level of methylation in H3K9 and H3K27 significantly decreases in CVD patients whereas methylation in H3K4, H3K9ac, and H3K27ac increases in atherosclerotic MSCs and macrophages. Another study by Greißel et al. [42,43,44] demonstrated that the histone acetyltransferase activity in GCN5-like protein 1 (GCN5L) and histone acetyltransferase KAT8 (MYST1) is correlated with the progression of atherosclerosis. In addition, they found that acetylation [45] histone acetyltransferase activity in GCN5L and MYST1 correlates with the progression of atherosclerosis. They also observed that acetylation in H3K9ac increases in the endothelial cells of atherosclerotic plaques. 

### 2.3. Aging-Related microRNAs (miRNAs)

The miRNAs are 19–25 nucleotides in length, encoded in the genome, and transcribed into primary miRNA (pri-miRNA) [46]. These small and unstable molecules regulate gene expression via degradation of the transcript or repression of translation when binding to the 3′-untranslated region of the target mRNA [47]. The expression levels of certain miRNAs are significantly upregulated during aging, leading to post-transcriptional inhibition of endothelial genes [48]. Numerous miRNAs have been described to be differently expressed and to regulate different cell types and pathways throughout cardiac aging (Table 1) [32,40,41]. Zhang et al., reported that cardiac miR-21 is upregulated with aging [36]. miR-21 has profibrotic effects that are induced via ERK–MAP kinase pathway activation in cardiac fibroblasts (CFs) after injury [37]. Cardiac miR-21 and miR-22 play a crucial role in heart aging and heart failure [36,49,50,51,52,53], whilst miR-29 promotes pathologic hypertrophy of cardiac myocytes and overall cardiac dysfunction. However, miR-29 is not an appealing target in older adults with heart failure since its overexpression could counteract post-myocardial infarction remodeling and otherwise sensitize the aorta to the formation of aneurysms (a common phenomenon during aging) [54,55]. miR-204 inhibition can modify vascular smooth muscle cell growth upon injury [56]. Upregulation of miR-122 leads to atherogenesis and endothelial dysfunction [57]. Similarly, the downregulation of other mRNAs is deleterious. One example is the downregulation of miR-181 during aging, CVD, and hypertension. These findings suggest that the loss of miR-181 is detrimental for the cardiovascular system [58,59,60]. Age-dependent NF-κB activation is associated with systemic inflammation and impaired endothelial-dependent vessel dilation [61,62,63,64,65,66,67] and, therefore, targeting miRs of this pathway is considered a potential therapy against proinflammatory cells (macrophages).

## 3. Aging, Epigenetic Modification, and Inflammation

The term “inflamm-aging” is a relatively new term added to the medical vocabulary by Franceschi et al., in 2000. It refers to the upregulation of the inflammatory response later in life as a consequence of epigenetic changes with a subsequent systemic low-grade chronic proinflammatory state that underlies most age-associated diseases [85,86]. One of the common effects of aging is the excessive production of inflammatory cytokines and reactive oxygen species (ROS) [58,63,64,67]. ROS production increases with age due to a variety of epigenetic stimuli, including physical, chemical, and biological agents. Oxidative stress occurs as an imbalance between ROS production and the body’s capacity to detoxify the resultant reactive intermediates or repair consequent impairment. ROS is behind endothelial dysfunction, effectively lowering the threshold for many diseases, especially CVD [87]. Oxidative stress increases vascular permeability and promotes leukocyte adhesion as well as an inflammatory response. A low level of chronic inflammation is associated with atherosclerosis, CVD, and diabetes. The immune system produces more proinflammatory cytokines under the regular stimulus accompanying aging. IL-6, TNF-α, and CRP mark the onset of CVD in older adults, and their levels correlate with the severity of left ventricular dysfunction and degree of activation of sympathetic and renin–angiotensin systems. Table 2 summarizes the most relevant epigenetic changes and inflammation processes in CVD. 

### 3.1. C-Reactive Protein (CRP)

CRP is a biomarker of systemic inflammation and a risk factor for the development of inflammation-mediated diseases such as CVD, metabolic syndrome, type 2 diabetes, and hypertension [88,89]. The production of CRP in the liver is triggered by cytokines (e.g., IL-6 which is secreted by macrophages and T cells) in response to inflammatory conditions. CRP level is associated with the epigenetic profile, specifically DNA methylation, which may represent the joint effect of both genetic and environmental factors [90]. Sun et al., identified over two hundred genes containing CRP-associated DNA methylation sites. The most significant CRP-associated DNA methylation sites are cg07073964, cg09358725, and cg11822932, which are in the KLK10, LIM, and LMO gene loci, respectively. There are several gene families related to the immune system that are enriched in the gene set of CRP-associated DNA methylation. Six immunoreceptor (CD) genes, CD1D, CD7, CD22, CD27, CD59, and CD82, and five interleukin and receptor genes, IL1R2, IL2RA, IL19, IL21R, and IL32, were identified by epigenetic association analysis. The methylation sites in five G-protein-coupled receptor (GPR) gene loci, GPR21, GPR65, GPR81, GPR84, and GPR171, were also found to be associated with CRP [90].

### 3.2. Interleukin 6 (IL-6)

IL-6 is a multifunctional cytokine that plays an important role in the development of ischemic heart diseases. DNA hypomethylation in the IL-6 promoter was associated with an increased risk for coronary heart disease, especially in acute myocardial infarction. Lepeule et al., suggested that differential DNA hypomethylation of the two distinct CpGs in IL-6 may reflect different cumulative effects from endogenous and exogenous exposure factors, and then contribute differently to the susceptibility to coronary heart disease. Transcription factor binding sites (BAF155, Inil, c-Myc, BAF170, Max, NRSF, and Nrf1) were identified for position 1, whereas position 2 was free of the binding sites [91].

### 3.3. Tumor Necrosis Factor α (TNF-α)

TNF-α is a proinflammatory cytokine with pleiotropic effects in human disease and well-characterized pathogenic contributions to inflammatory and autoimmune diseases such as atherosclerosis and type 2 diabetes. Treatment with TNF inhibitors has been shown to lower the risk of cardiovascular disease among patients with autoimmune disease [92]. Altered methylation of CpG loci in the TNF promoter has been associated with TNF-α expression [93,94]. In addition, DNA methylation loci in two genomic regions mapping to NLRC5 and DTX3L/PARP9 changes expression of corresponding genes and alters circulating TNF-α levels. These processes are induced chiefly by interferon γ (IFN-γ) stimulation, Toll-like receptor ligands, and other interferons in response to diverse stimuli such as viral infections [95]. By activating CD8+ T cells via major histocompatibility complex class I proteins, NLRC5 has also been shown to upregulate IFN-γ, creating a positive feedback loop that ensures an effective response to intracellular pathogens [96]. Increased expression of DTX3L-PARP9 has been shown to enhance IFN-γ signaling and therefore host immune response [97]. Recent evidence suggests that DTX3L-PARP9 may also play a key role in vascular inflammation and atherosclerosis. There are associations between TNF-α levels and methylation loci in the α 1-3-n-acetylgalactosaminyltransferase, and α 1-3-galactosyltransferase gene (ABO) [98]. 

## 4. Effect of Lifestyle and Environmental Factors on Epigenetic Modification in Older Adults with CVD

**Figure 1:**
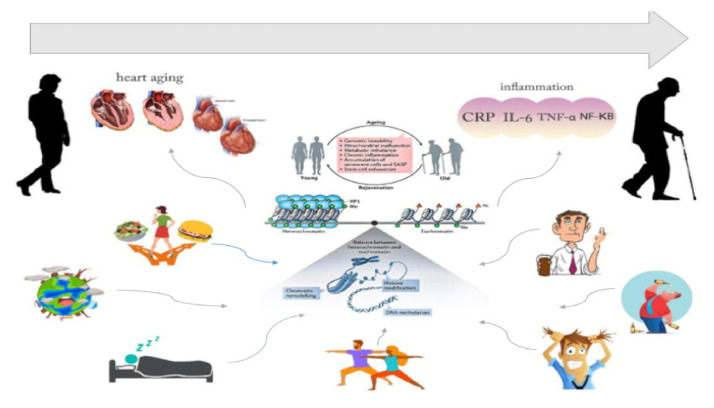
Interaction between various lifestyle interventions on epigenetic alterations and CVD in older adults.

Lifestyle and environmental factors contribute to epigenetic modifications with a cumulative effect during aging. These factors seemed to accelerate the aging process and affect health by triggering age-related chronic illnesses such as vascular aging [106,107]. Genome-wide association studies and epigenome-wide association studies highlighted the crucial role of diet in the development of chronic diseases associated with aging. Epigenetic mechanisms favor the development of obesity and type 2 diabetes which are important risk factors for CVD [108]. Both conditions are prevalent in older adults and totally dependent on lifestyle variables [100]. The well-known lifestyle and environmental factors related to epigenetic modification and aging in CVD patients are summarized in Table 3 and Figure 1.

### 4.1. Nutritional Habits and Food Consumption

#### 4.1.1. Nutrients

Several studies have focused on dietary patterns and their effects on risk of disease and mortality. For example, the Mediterranean diet is associated with a lower degree of inflammation and has a protective role on cardiovascular and cerebrovascular events. Olive oil, which is abundant in the Mediterranean diet, contains phenols, which have anti-inflammatory effects that can help to reduce the risk of cancer. Some of the beneficial effects of olive oil have been attributed to its epigenetic effects, and in particular its effects on DNA methylation pattern [109,110]. A clinical trial with individuals with class II/III obesity showed that 50 mL/day extra virgin olive oil modulate positive changes in body composition, reducing body fat and increasing lean mass and free fat mass, in addition to the presence of the Ala allele of the Pro12Ala polymorphism [111].

A recent genome-wide DNA methylation study with 3096 participants demonstrated that tea and coffee consumption are also associated with altered methylation in two differentially methylated CpG sites (DNAJC16 and TTC17) [111]. Another study reported an association between ω-3 PUFA supplementation and vegetable and fruit consumption and lower GrimAgeAccel, DNAm PAI-1, DNAm ADM, and DNAm cystatin C which are considered epigenetic age markers enriched for DNA methylation sites that are surrogate biomarkers for blood plasma proteins related to aging [112,113].

It is well known that fatty acids have a distinct influence on DNA methylation. In an in vitro study with arachidonic acid (AA) and oleic acid (W9) [114,115], they provided complex results characterized by a general hypermethylation and hypomethylation of the DNA, respectively. AA-coordinated DNA methylation occurred for palmitic acid, atherosclerosis, diabetes, and obesity. Evidence indicates that β-oxidation of the peroxisome proliferator activated receptor alpha (PPAR-α) and sirtuin 1 is essential to mediate DNA methylation changes caused by AA [115].

Cruciferous vegetables such as broccoli, cauliflower, cabbage, kale, watercress, and Brussels sprouts contain sulforaphane, which is an isothiocyanate which can alleviate age-related diseases. This effect is due, in part, to the inhibition of inflammation by hypomethylation of the DNA. For example, the attenuation of DNA hypermethylation was mediated by DNMT in the promoter region of factor 2 related to NF-E2 (Nrf2), noting that Nrf2 is a transcription factor that regulates the reduction–oxidation balance and has been linked to inflammation and neurodegeneration [109,116].

#### 4.1.2. Phytochemicals and Representative Compounds

Curcumin has long been used not only as a very common spice in Eastern cuisine but also as a medicinal herb. It is a phytochemical that has been shown to improve metabolic dysfunction. Studies indicate that curcumin is metabolically effective in regulating DNA methylation. Li et al., demonstrated that curcumin reduced DNA hypermethylation at CpG sites [C0] 360, [C0] 341, [C0] 329, [C0] 316, and [C0] 307 in the PPARα promoter region [109,117,118].

Ascorbic acid is known as a potent antioxidant and anti-inflammatory. It attenuates “inflammation”, regardless of its antioxidative function, because it has been shown to modify the epigenome. Ascorbate has been identified as a cofactor for the methylcytosine dioxygenases that are responsible for DNA demethylation. It is also a potential cofactor for some JmjC field containing histone demethylases that accelerate histone demethylation. Therefore, ascorbic acid can combat a proinflammatory state of aging, and its consequent diseases, regulating the function of immune cells that require the activation of demethylase [119,120,121].

#### 4.1.3. Trace Elements and Vitamins

Similarly, magnesium and selenium levels may function as potential epigenetic regulators via modulating different signaling pathways [112,113,122]. The possible epigenetic effects of selenium are the modulation of epigenetic information editors, interaction with miRNAs, as well as influence on the metabolism of a carbon, which acts as a methyl donor for DNA methylation [123,124].

Fiorito et al. [125] reported that a low dietary intake of B vitamins promotes DNA methylation in specific genes (TCN2, CBS, PON1, AMT) that mediate CVD risk. In addition, a low intake of riboflavin has been associated with higher methylation at 1 CpG (cg21230392) [51,92,96,112], and supplementation with flavanols and polyphenols may affect the activity of DNMTs [112,126]. Higher LINE-1 methylation levels have been demonstrated in participants of the North Texas Health study who had high vegetable and fruit intake [126].

### 4.2. Physical Activity

Epigenetic mechanisms may be implicated in mediating the effects of physical activity [110]. Physical activity can modulate gene expression through epigenetic alternations. Physical activity causes epigenetic effects that can result in health benefits and help prevent chronic diseases. The different effects related to the type, duration, and intensity of physical activity are still unclear. The epigenetic mechanisms that interact with physical activity collaborate to reduce basal inflammation, thus preventing the development of diseases associated with low-grade chronic inflammation, such as obesity and diabetes. It is already known that physical activity attenuates hypomethylation and hypermethylation processes linked to neoplastic mutations in the genome, in addition to post-translational modifications of histones (hPTMs) and non-coding RNA, especially microRNAs (miRNAs), can be induced by physical activity [106,127].

### 4.3. Smoking

Tobacco smoking induces dysregulated DNA methylation in hundreds of CpG sites which are related to the epigenetic clock [110]. Therefore, the epigenetic alterations of smoking and vaping include DNA methylation, microRNA, and non-coding RNA, and research in animals and humans has also reported that the use of electronic cigarettes (vaping) is linked to worse general and respiratory health, similar to effects observed with conventional smoking [128].

### 4.4. Alcohol Consumption

It is well established in the literature that ethanol can alter gene expression through epigenetic mechanisms, that is, prolonged exposure to ethanol can alter DNA and histone methylation, histone acetylation, and microRNA expression [129].

### 4.5. Psychological Stress and Insufficient Sleep

Psychological stress and epigenetic aging have significant associations and primary findings propose that epigenetic aging could be avoidable and in some cases reversible [130]. A randomized prevention trial by Brody et al., to test hypotheses about the ways risky family processes contribute to accelerated epigenetic aging suggested that developmentally appropriate family-centered interventions designed to enhance parenting and strengthen families can buffer the biological residue of life in a risky family. The intervention ameliorated the effect of parental behavior on offspring epigenetic aging.

New findings suggest that appropriate targeting of behavioral interventions might prevent the influence of a stressful environment on the aging process. A cohort study showed that changes in epigenetic factors and aging were associated with different stress-related measures within a 6-month interval. For example, results obtained by Boks et al. [131] demonstrated that exposure to war in Afghanistan was associated with increased DNA methylation-predicted age.

Insufficient sleep may have an impact on epigenetics. An epigenetic reprogramming of circadian genes, changes in Alu repetitive element methylation, and gene-specific methylation of IFN-γ and TNF-α promoters have been observed [110]. Short sleep duration or insomnia may lead to an increased risk of various psychiatric and cardio-metabolic conditions. A cross-sectional genome-wide analysis of DNA methylation by Lahtinen et al., demonstrated a strong relationship between self-reported insufficient sleep and that epigenetic modifications in ERC2, MAGI2, CAST, and CDK5R1 might be triggered by insufficient sleep [132].

### 4.6. Environmental Factors

#### 4.6.1. Arsenic

Observational studies have shown that exposure to arsenic is associated with hypo- and hypermethylation at various genetic loci in vivo or in vitro [133].

#### 4.6.2. Air Pollution

Air pollution exposure is estimated to contribute to approximately seven million early deaths every year worldwide and more than 3% of disability-adjusted life years lost [134] (REF). Air pollution has numerous harmful effects on health and contributes to the development and morbidity of cardiovascular disease, metabolic disorders, and several lung pathologies. Emerging data indicate that air pollution exposure modulates the epigenetic DNA methylation, and that these changes might in turn influence inflammation, disease development, and exacerbation risk [135,136,137].

#### 4.6.3. Aromatic Hydrocarbons and Other Organic Pollutants

These environmental pollutants are a potential risk for morbidity and mortality in the community in general and in workers exposed to them. A study in an animal model showed that exposure to polycyclic aromatic hydrocarbons favors the non-monotonic modification of (hydroxy)methylation of DNA and RNA, in addition to affecting the glutathione status [138].

#### 4.6.4. Shift Work

DNA methylation can be used to assess biological age which has been associated with age-related disease risks and is strongly influenced by shift work. A study of 2574 non-Hispanic white women aged 35–74 was carried out to quantify the biological consequences of shift work. The greatest acceleration of age was observed in night work (β = 0.12, 95% CI: 0.03–0.21). The association was stronger for ≥10 years of night work (β = 3.16, CI 95%: 1.17–5.15), and when evaluating the association of the whole epigenome, years of night work were associated with DNA methylation at 85 CpG sites (FDR < 0.05). Years of night work were associated with lower methylation of CpG in the gene body of ZFHX3 (cg04994202, q = 0.04), a gene linked to the circadian rhythm [139,140].

Recent evidence shows that lifestyle and environmental factors may affect epigenetic mechanisms, such as DNA methylation, histone acetylation, and miRNA expression. Several common substances or nutrients such as caffeine (present in coffee and some teas) and curcumin or trace elements like selenium and magnesium could regulate epigenetics. Similarly, physical inactivity, alcohol consumption, air pollutants, psychological stress, and shift working are well-known modifiers of epigenetic patterns. Understanding the exact ways that lifestyle and environmental factors could affect the expression of genes could help to influence the time of incidence and severity of aging-associated diseases.

## 5. Conclusions

Aging is a natural and unavoidable process that is associated with epigenetic changes that may impair tissue and organ function. Epigenetic effects are manifested by altered gene transcription in response to lifestyle and environmental cues and provide mechanistic insight into individual responses to the environment. Ensuring healthy aging requires lifestyle modifications that counteract deleterious effects of epigenetic alterations such as adequate physical activity and promoting healthy nutrition. In the last few years, several investigations have examined the relationship between epigenetic marks and lifestyle factors, including nutrition, behavior, stress, physical activity, working and sleep habits, smoking, and alcohol consumption. These studies have shown that a healthy diet (rich in fibers, vitamins, essential elements, and beneficial fatty acids), adequate physical activity levels, smoking cessation, stress reduction, and adequate sleep could be useful in preventing deleterious epigenetic changes that lead to the activation of inflammatory processes. Although epigenetic modifications are influenced by lifestyle and the environment and are mostly modifiable, they may be transmittable from one generation to another [161]. There is a possibility that this phenomenon impacts successive generations it is referred to as transgenerational epigenetic inheritance [162,163]. Further research into the role of epigenetics will assist in better understanding healthy aging and avoiding early aging.

## Figures and Tables

**Table 1:** Summary of microRNA (miRNA) involvement in cardiac and vascular aging.

Tissue	miRNA		Molecular Targets	Functions
Aging heart	miR-21 [68]	Increase	ERK-MAP kinase signaling	Profibrotic (role on cardiac fibroblast (CFS))
Aging heart	miR-22 [69]	Increase	Mimecan/osteoglycin	Accelerate CF senescence and migration
Aging heart	miR-18 and miR-19 [70]	Decrease	Thrombospondin-1 and connective tissue growth factor	Anti-heart failure-related fibrosis during aging
Aging heart	miR-17-3P [71]	Decrease	PAR-4	Reduce CF cellular senescence
Aging heart	miR-34a [72]	Increase	Phosphatase 1 nuclear targeting subunit	Increase age-related cardiomyocyte apoptosis and cardiac dysfunction
Vascular aging	miR-34a [73]	Increase	SIRT1	Stimulate senescence in endothelial cells and vascular smooth muscle cells
Vascular aging	miR-217 [74]	Increase	SIRT1	Stimulate endothelial senescence, decrease nitric oxide
Vascular aging	miR-29 [75]	Decrease	Collagen and elastin	Extracellular matrix impairment (risk of age-related aortic aneurism)
Vascular aging	miR-146 [76]	Increase	IRAK and NOX4	Proinflammation or antioxidative stress
Vascular aging	miR-92 [77]	Decrease	TNF receptor 1 and collagen type1	Reduction in mimic arterial aging
Vascular aging	mir-20a [78]	Decrease	MKK3, activation of p38 MAP kinase	Inhibit endothelial cell migration by the inhibition of MKK3 and the activation of p38 MAP kinase
Vascular aging	mir-126 [79]	Decrease	PLGF	Increase cell apoptosis, decrease proliferation, endothelial cell migration
Vascular aging	mir-10a [80]	Decrease	p53/Rb network, including p53 regulator MDM4, Rb regulator RB1CC1, p21 regulator TFAP2C, p53	Endothelial progenitor cell dysfunction
Vascular aging	mir-21 [81]	Decrease	PTEN, SPRY1, SPRY2	Regeneration of endothelial progenitor cells
Vascular aging	mir- 217 [82]	Increase	SIRT1, FOXO1, eNOS	Impair endothelial angiogenesis
Vascular aging	mir-146a [83]	Decrease	Toll-like receptor 4 (TLR4)	Senescent endothelial cells
Vascular aging	mir-17-92 [84]	Decrease	Chk1/2, G-H2AX, ATM	Regulators of chromatin-related proteins
Vascular aging	miR-204-3p [56]	Decrease	PDGF	Vascular smooth muscle cell growth upon injury

**Table 2:** Summary of the most relevant epigenetics changes and inflammation processes in CVD.

Epigenetic Modifications	Sites	Affected Gene
DNA methylation	KLK10, LIM, LMO, D1D, CD7, CD22, CD27, CD59 and CD82, IL1R2, IL2RA, IL19, IL21R, IL32, GPR21, GPR65, GPR81, GPR84, and GPR171	CRP [99]
	BAF155, Inil, c-Myc, BAF170, Max, NRSF, and Nrf1	IL-6 [100]
	NLRC5 and DTX3L/PARP9, IFN-γ, and ABO	TNF-α [101]
Histone modification	H3K4me3	SIRT1, FoxO3, NF-κB, and p53 [102] TNF-α [103], SET1A/B, SET7, MLL1/2, MLL3/4, LL1, and VEGFA [104]
	H3K4me3 and H3K9ac	TNF-α [103]
	H3K9me2	VSMC [105]

**Table 3:** Lifestyle and environmental factors and type of epigenetic modification and CVD.

Item		Epigenetic Changes			
		DNA Methylation	Histone Modification	microRNA Involvement	DNMT Enzymes
Nutritional habits and food consumption	Polyunsaturated fatty acids [141]	+			
	Arachidonic acid and oleic acid [142]	+			
	Diets rich in fruits and vegetables—sulforaphane— for example, broccoli, cauliflower, cabbage, kale [143]	+			+
	Folate and vitamin B12 intake [121]	+	+	+	+
	Polyphenols (green tea, tea, and coffee are a rich source of polyphenols) [144]		+		-
	Magnesium and selenium [122]	+	+		
	Curcumin	+			
	Ascorbic acid	+			
Physical activity [145,146]		+	+	+	
Tobacco smoke [147,148,149]		+	+	+	
Alcohol consumption [110]		+			
Psychological stress and sleep deficiency [132,150,151,152,153,154,155]		+			
Environmental pollutants	Arsenic [156,157,158]	+			
	Air pollution [159]	+		+	
	Aromatic hydrocarbons and other organic pollutants	+			
Shift work [160]		+			
